# SMAC is expressed *de novo *in a subset of cervical cancer tumors

**DOI:** 10.1186/1471-2407-4-84

**Published:** 2004-11-23

**Authors:** Magali Espinosa, David Cantu, Carlos M Lopez, Jaime G De la Garza, Vilma A Maldonado, Jorge Melendez-Zajgla

**Affiliations:** 1 Subdirección de Investigación Básica. Instituto Nacional de Cancerología. Av. San Fernando # 22. Tlalpan 14080 México, D.F. MEXICO

## Abstract

**Background:**

Smac/Diablo is a recently identified protein that is released from mitochondria after apoptotic stimuli. It binds IAPs, allowing caspase activation and cell death. In view of its activity it might participate in carcinogenesis. In the present study, we analyzed Smac expression in a panel of cervical cancer patients.

**Methods:**

We performed semi quantitative RT-PCR on 41 cervical tumor and 6 normal tissue samples. The study included 8 stage I cases; 16 stage II; 17 stage III; and a control group of 6 samples of normal cervical squamous epithelial tissue.

**Results:**

Smac mRNA expression was below the detection limit in the normal cervical tissue samples. In contrast, 13 (31.7%) of the 41 cervical cancer biopsies showed detectable levels of this transcript. The samples expressing Smac were distributed equally among the stages (5 in stage I, 4 in stage II and 4 in stage III) with similar expression levels. We found no correlation between the presence of Smac mRNA and histology, menopause, WHO stage or disease status.

**Conclusions:**

Smac is expressed *de novo *in a subset of cervical cancer patients, reflecting a possible heterogeneity in the pathways leading to cervical cancer. There was no correlation with any clinical variable.

## Background

Apoptosis is an evolutionarily conserved biological process that plays a fundamental role in development and tissue homeostasis in metazoans [1]. This type of cell death is executed by a family of proteases known as caspases [2]. There are two well-characterized apoptotic pathways that converge in caspase activation: the death receptor pathway and the mitochondrial pathway [3]. Inhibitors of Apoptosis Proteins (IAPs) are the most important regulators of caspases. These proteins inhibit caspase activation, thus preventing the induction of apoptosis [4]. In cells undergoing apoptosis, IAPs are inactivated by interaction with proteins containing the so-called IBM (IAP-binding motif) [4,5].

One IBM protein is the recently identified Smac/DIABLO [6,7]. Smac resides in the mitochondrial intermembrane space in healthy cells but is released into the cytosol during apoptosis, where it interacts with IAPs and disrupts their ability to bind caspases [8]. Smac is expressed ubiquitously, with high expression in adult testis, heart, liver, kidney, spleen, prostate and ovary and low expression in brain, lung, thymus, and peripheral blood leukocytes [9]. It is encoded in a nuclear gene and is post-translationally imported into the mitochondria via a targeting sequence in its amino terminus. Removal of this signal generates a mature polypeptide with the IBM at the amino terminal end [10]. Smac interacts with all mammalian IAPs examined so far: XIAP, cIAP-1, cIAP-2, survivin and ML-IAP [6,7,11,12]. The structure of the Smac-XIAP complex has been studied by X-ray crystallography [13] and high-resolution NMR [14]; it appears that the tetrapeptide AVPI is indispensable for the formation of this complex.

IAPs are highly expressed in human tumor cells [15-17], contributing to the intrinsic resistance of these cells to endogenous death receptor-induced apoptosis and consequently to chemotherapy [18]. For this reason, peptides mimicking the action of Smac have been generated and analyzed. Four publications to date have shown promising effects of these Smac peptides *in vitro *and *in vivo*; however, further studies are required prior to clinical testing [19-22].

Recently, Sekimura and colleagues found that Smac expression was significantly lower in primary lung cancers than in normal tissue [23]; patients with lower Smac mRNA levels had worse prognoses. These results indicate that Smac expression may play a role in the progression of primary lung cancer and may be useful for prognosis [23]. However, Smac expression has not been analyzed in other tumors. In view of the possible role of Smac in cervical carcinogenesis and its potential as a therapeutic target, we have investigated the expression of this apoptotic protein in cervical cancer patients.

## Methods

### Cell lines and tumor samples

Cervical cancer cell lines (HeLa, SiHa, CaSki and CaLo) were obtained from ATCC and cultured as monolayers in Dulbecco Modified Eagle's Medium (DMEM) containing 10% (V/V) fetal bovine serum (GIBCO, Bethesda, MD, USA) at 37°C in a humidified atmosphere of 5% (V/V) CO_2_.

Forty-one cervical cancer samples were obtained from the Instituto Nacional de Cancerologia of Mexico. Written consent was obtained from patients before the samples were collected. Tumors were staged according to the International Gynecology and Obstetric Federation (FIGO) system. The samples comprised 8 at stage IB, 16 at stage IIB and 17 at stage IIIB; and a control group comprising 6 samples of normal cervical squamous epithelial tissue (Table 1). The control samples were derived from hysterectomy specimens from patients with uterine myomatosis. Only samples with normal pathological reports were included.

### Histology

Histopathological grading was done according to the WHO (World Health Organization) classification system (Table 1).

### RNA isolation and RT-PCR

RNA extraction and RT-PCR analysis were performed as described previously [24]. Briefly, total RNA was extracted from cultured cells, tumors and non-neoplastic tissue samples with Trizol reagent (Invitrogen) following the manufacturer's protocol. RNA purity was confirmed by the 260/280 nm absorbance ratio and its integrity was established with agarose gels. Total RNA (2 μg) was reverse-transcribed in a final 20 μl reaction volume using 15 U ThermoScript reverse transcriptase, 2.5 × RT Buffer and random hexamers (ThermoScript RT-PCR, Invitrogen). The RT-PCR steps were 25°C for 10 min, 50°C for 50 min and 85°C for 5 min. Smac and GAPDH mRNA PCR reactions contained 0.25 μl Amplitaq gold polymerase (Applied Biosystems, ROCHE), 2.5 μl 10 × reaction buffer, 0.5 μl dNTP mix 10 mM, 1 μl sense primer 10 μM, 1 μl anti-sense primer 10 μM and 1 μl cDNA in 25 μl final volume. The Smac primers were: sense 5' GCGCGGATCCATGGCGGCTCTGAAGAGTTG 3' and anti-sense 5' AGCTCTCTAGACTCAGGCCCTCAATCCTCA 3'. The GAPDH primers were: sense 5' CCCCTTCATTGACCTCAACT 3' and antisense 5' TTGTCATGGATGACCTTGGC 3'.

The PCR cycle parameters for Smac were: 10 min enzyme activation at 95°C followed by 3 cycles of 30 s at 95°C and 2 min at 72°C, then 30 cycles of 30 s at 95°C and 30 s at 68°C, and finally 5 min at 72°C. The corresponding parameters for GAPDH were: 10 min enzyme activation at 95°C followed by 25 cycles of 30 s at 95°C, 30 s at 60°C and 30 s at 72°C. The products were electrophoresed on 1% agarose gels and stained with ethidium bromide. Smac mRNA data were expressed as ratios between the densitometric values (Scion Image software) of Smac gene expression. The PCR products were normalized to the amplified GAPDH, the internal reference gene. Gene expression measurements were repeated at least twice.

### Statistical analysis

To detect a correlation between pathological tumor parameters and normalized Smac expression we used ANOVA (stage, current disease and menopause status) and *chi *square tests (stage, histology of tumors, menopause and current status). Kaplan-Meier curves for status were generated and log rank was used to test for differences. The mean follow-up was 14.7 months. The statistical package Intercooled Stata 7.0 was used for analyses and statistical significance was accepted when the *p *value was less than 0.05.

## Results

**Figure 1:**
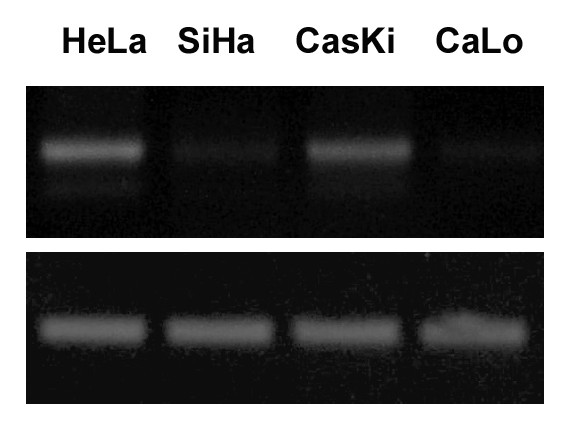
Smac/Diablo mRNA expression in cervical cancer cell lines. Upper panel: RT-PCR analysis of HeLa, SiHa, CasKi and CaLo cervical cancer cell lines. To the left molecular weight marker (100 bp ladder, Invitrogen). Lower panel: RT-PCR of GAPDH, used as a mRNA load control.

To ascertain whether Smac is expressed in cervical cancer we performed semiquantitative RT-PCR analyses on a panel of cervical cancer lines, including HeLa, SiHa, CasKi and CaLo cells. As shown in Figure 1, the HeLa and CasKi lines contained Smac mRNA, but very low levels were observed in SiHa and CaLo cells.

**Figure 2:**
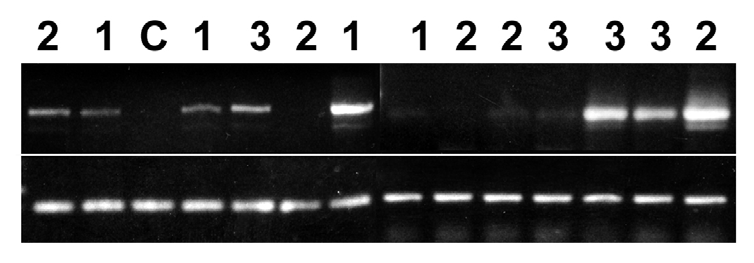
Smac/Diablo mRNA expression in cervical cancer patients. Upper panel: RT-PCR analysis of Smac/Diablo mRNA. To the left molecular weight marker (100 bp ladder, Invitrogen). Clinical stage is showed at the top of the panel: C: control samples, 1, 2 and 3, clinical stages. Lower panel: RT-PCR of GAPDH, used as a load control.

**Figure 3:**
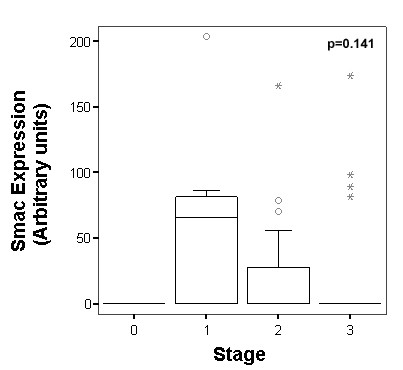
Smac expression levels versus clinical stage of cervical cancer samples. Graph shows median, upper and lower quartiles. P value testing the significance of the difference by ANOVA.

**Figure 4:**
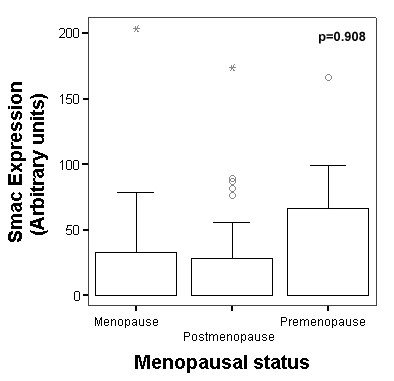
Smac expression levels versus menopausal status of cervical cancer samples. Graph shows median, upper and lower quartiles. P value testing the significance of the difference by ANOVA.

**Figure 5:**
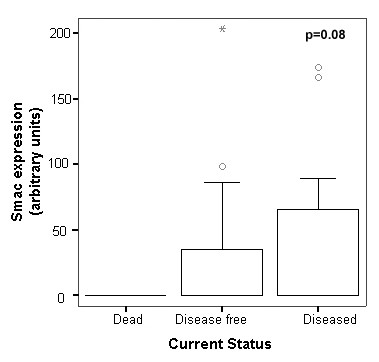
Smac expression versus disease status of cervical cancer samples. Graph shows median, upper and lower quartiles. P value testing the significance of the difference by ANOVA.

**Figure 6:**
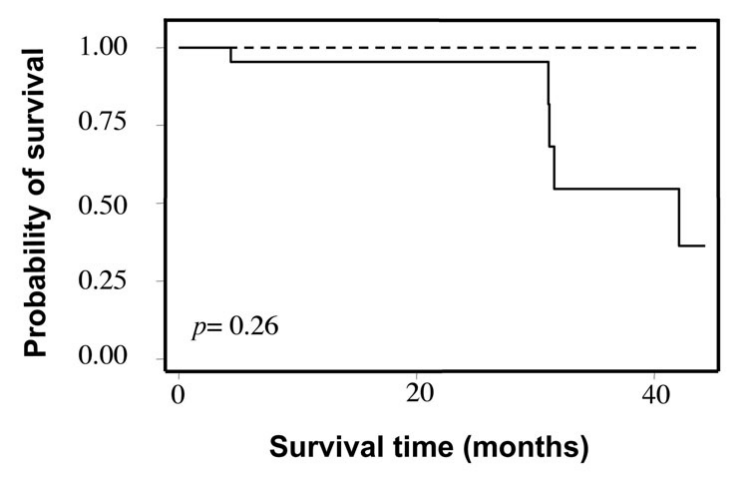
Kaplan/Meier survival analysis of cases by Smac expression. Continue black line: Negative expression. Dotted line: Positive expression. Insert in the lower left corner of plot is the P value testing the significance of the difference in the survival curves by the Mantel/Cox log rank test

Next, we measured Smac mRNA levels using the same approach in 41 cervical tumor and 6 normal cervical samples. To ensure accurate determinations and to verify equal RNA input, GAPDH mRNA was amplified simultaneously. Figure 2 shows a representative panel of results, which are given in Tables 1 and 2. Unexpectedly, Smac mRNA was below the detection limit in normal cervical samples. In contrast, as expected from the cell line data, 13 (31.7%) of the 41 cervical cancer biopsies contained detectable levels of this transcript. The samples expressing Smac were distributed equally among the stages (5 in stage I, 4 in stage II and 4 in stage III). We found no significant correlation between Smac mRNA level and histology, menopause, clinical stage or disease status (Table 2). When the Smac expression levels in the tumor samples were analyzed, there were no significant differences between clinical stages (Figure 3), menopause status (Figure 4) or disease status (Figure 5). Similarly, a survival analysis of the patients showed no statistical differences between patients expressing or not expressing Smac mRNA (Figure 6).

## Discussion

Tumors proliferate beyond the constraints that limit growth in normal tissue. Therefore, the resistance of tumor cells to apoptosis is an essential feature of carcinogenesis. This has been confirmed by the finding that deregulated proliferation alone is not sufficient for tumor formation because there is concomitant induction of cell death [25]. Overexpression of growth-promoting oncogenes such as c-Myc sensitize cells to apoptosis [26]. Thus, tumor progression requires the expression of anti-apoptotic proteins or the inactivation of essential pro apoptotic proteins [27,28]. Indeed, it has been shown that survivin, a member of the Inhibitor of Apoptosis Protein (IAP) family, is upregulated in some tumors [29], correlating with prognosis [30,31].

Smac is a recently identified proapoptotic protein that interacts with and inhibits several IAPs, including survivin [6,11]. It has been shown that Smac mRNA levels in tumor tissues are significantly lower than in normal tissues [23]. Patients with lower Smac mRNA levels have worse prognoses. These results indicate that Smac expression may play a role in the progression of primary lung cancer, as expected by the known role of this protein in cell death induced by chemotherapeutic drugs. Unexpectedly, we found that during cancer progression, some cervical tumors express this protein *de novo*.

Unfortunately, we found no correlation between Smac expression and any clinical variable. This could be attributed to differences in tissue expression of IAPs, which are reported to have different binding affinities for Smac. On the other hand, alternative IAPs such as the recently identified Omi/Htra2 [32] might play an important tissue- or tumor-specific role. This is supported by the recent report of a null phenotype in Smac-deficient mice, in which a role for other IAP inhibitory proteins is suspected [33].

Cancer treatment by chemotherapy and γ-irradiation kills cells primarily by the induction of apoptosis. However, few tumors are wholly sensitive to these therapies, and the development of resistance to therapy is an important clinical problem. Failure to activate the apoptotic programme represents an important mode of drug resistance in tumor cells [34]. Modulation of the key elements in apoptotic signaling should directly influence therapy-induced tumor-cell death. Indeed, it has recently been suggested that peptides mimicking the Smac amino-terminus could be a novel therapeutic weapon [19]. Tumors with low or null Smac expression, such as the ones reported in this study, could be more susceptible to this approach.

## Conclusions

During cervical cancer progression, a subset of tumors express the apoptotic protein Smac *de novo*. This finding contrasts with a previous report for lung cancer [23], underlining the notion that downregulation or even expression of Smac could be dispensable for tumor progression, at least in cervical cancer. This could be because other mitochondrial molecules such as Omi might substitute for its known proapoptotic function. There was no correlation between Smac expression and any clinical variable.

## Competing interests

The author(s) declare that they have no competing interests.

## Authors' contributions

JMZ Conceived and coordinated the study.

VAML: Conceived and coordinated the study. Statistical Analysis

MEC: Performed RT-PCR assays

DCL: Provided the clinical samples and coordinated patient study

CMLG: Coordinated patient assessment, ethical guidelines.

JGGS: Provided clinical assessment

## Pre-publication history

The pre-publication history for this paper can be accessed here:



## Figures and Tables

**Table 1:** Smac mRNA expression levels and clinicopathological factors in cervical cancer

***Sample***	***Age***	***Stage***	***Histology of tumor***	***Menopause***	***Current status***	Smac/***GAPDH***
Control	42		-	Menopause	Disease-free	0
Control	33		-	Pre-menopause	Disease-free	0
Control	28		-	Pre-menopause	Disease-free	0
Control	44		-	Menopause	Disease-free	0
Control	47		-	Menopause	Disease-free	0
Control	35		-	Pre-menopause	Disease-free	0
1	46	I	Adenocarcinome	Menopause	Disease-free	0
2	52	I	Squamous cell	Post-menopause	Disease-free	76.01
3	49	I	Squamous cell	Menopause	Disease	203.36
4	44	I	Squamous cell	Pre-menopause	Disease-free	0
5	65	I	Squamous cell	Post-menopause	Disease-free	0
6	67	I	Squamous cell	Post-menopause	Disease-free	86.53
7	47	I	Squamous cell	Menopause	Disease-free	65.3
8	34	I	Squamous cell	Pre-menopause	Disease-free	66.45
9	49	II	Squamous cell	Menopause	Disease-free	78.44
10	63	II	Squamous cell	Post-menopause	Disease-free	0
11	38	II	Squamous cell	Menopause	Disease-free	0
12	48	II	Squamous cell	Menopause	Disease-free	0
13	55	II	Squamous cell	Post-menopause	Disease-free	0
14	35	II	Squamous cell	Pre-menopause	Disease-free	166.25
15	66	II	Squamous cell	Post-menopause	Disease	0
16	52	II	Squamous cell	Post-menopause	Disease	0
17	80	II	Squamous cell	Post-menopause	Disease	0
18	70	II	Squamous cell	Post-menopause	Disease-free	0
19	65	II	Squamous cell	Post-menopause	Disease	0
20	39	II	Squamous cell	Pre-menopause	Disease	70.42
21	57	II	Squamous cell	Post-menopause	Disease-free	55.45
22	37	II	Squamous cell	Pre-menopause	Disease-free	0
23	59	II	Squamous cell	Post-menopause	Disease	0
24	36	II	Squamous cell	Pre-menopause	Disease	0
25	33	III	Adenocarcinome	Pre-menopause	Dead	0
26	50	III	Adenocarcinome	Post-menopause	Dead	0
27	60	III	Squamous cell	Post-menopause	Disease-free	0
28	64	III	Adenocarcinome	Post-menopause	Dead	0
29	80	III	Squamous cell	Post-menopause	Dead	0
30	52	III	Squamous cell	Post-menopause	Disease-free	173.96
31	56	III	Squamous cell	Post-menopause	Disease-free	0
32	70	III	Squamous cell	Post-menopause	Disease-free	81.5
33	72	III	Adenosquamous	Post-menopause	Dead	0
34	33	III	Squamous cell	Pre-menopause	Disease	0
35	82	III	Adenocarcinome	Post-menopause	Disease	0
36	48	III	Squamous cell	Menopause	Disease-free	0
37	32	III	Squamous cell	Pre-menopause	Disease	98.59
38	48	III	Squamous cell	Menopause	Disease	0
39	36	III	Adenosquamous	Pre-menopause	Disease	0
40	52	III	Squamous cell	Post-menopause	Disease-free	0
41	67	III	Squamous cell	Post-menopause	Disease-free	88.95

**Table 2:** Smac positivity in cervical cancer tumor samples.

**Variable**	No. Patients (n = 41)	Smac Positive (n = 13)	Smac Negative (n = 28)	***P***
**Age**	53.36 (32–82)	50	54.92	0.3
**Stage**				
IB	8	5	3	
IIB	16	4	12	0.11
IIIB	17	4	13	
**Histology of tumors**				
Squamous cell	34	13	21	
Adenocarcinoma	5	0	5	0.14
Adenosquamous	2	0	2	
**Menopausal status**				
Pre-Menopause	12	5	7	
Menopause	6	2	4	0.64
Post-Menopause	23	6	17	
**Current status**				
Disease free	21	9	12	
Diseased	15	4	11	0.15
Dead	5	0	5	
